# Patterns of Cognitive Impairment in Hemodialysis Patients and Related Factors including Depression and Anxiety

**DOI:** 10.3390/jcm12093119

**Published:** 2023-04-25

**Authors:** Aleksandra Golenia, Norbert Żołek, Piotr Olejnik, Paweł Żebrowski, Jolanta Małyszko

**Affiliations:** 1 Department of Neurology, Medical University of Warsaw, 02-097 Warsaw, Poland; 2 Institute of Fundamental Technological Research, Polish Academy of Sciences, 02-106 Warsaw, Poland; 3 Department of Nephrology, Dialysis and Internal Diseases, Medical University of Warsaw, 02-097 Warsaw, Poland

**Keywords:** hemodialysis, cognitive impairment, depression, anxiety

## Abstract

Introduction: Hemodialysis patients are at higher risk of developing cognitive impairment, but the pattern of affected cognitive domains is still undetermined. Little is also known about the symptoms of depression and anxiety in hemodialysis patients. Methods: In this cross-sectional study, we included 74 consecutive adult patients undergoing hemodialysis. Cognitive functions were assessed using the Addenbrooke Cognitive Test III. In addition, all patients were screened for symptoms of depression and anxiety using the Hospital Anxiety and Depression Scale. Results: The mean age of hemodialysis patients was 65.69 ± 14 years. Among the patients, there were 27% and 31% of patients with mild cognitive impairment and suspected dementia, respectively. In the group of patients with suspected dementia, all cognitive functions had significantly lower values compared to these functions in incognitively unimpaired and mild cognitive impairment patients. The most impaired domain was verbal fluency, which reflects impairments in executive function. Depression and anxiety symptoms were observed in 28% and 22% of patients, respectively. Patients with anxiety symptoms had higher levels of endogenous creatinine, parathyroid hormone, and hemoglobin, as well as decreased creatinine clearance, being younger and less educated. No factors contributing to the occurrence of depressive symptoms were found. Conclusion: Cognitive dysfunction is a significant problem in hemodialysis patients. Our study showed that the prevalence of cognitive impairment and depression and anxiety symptoms in hemodialysis patients was high. The domain of executive functions was most affected. Furthermore, creatinine, parathyroid hormone, hemoglobin levels, creatinine clearance, and education affected the anxiety scale score.

## 1. Background

End-stage renal disease (ESRD) is a growing problem worldwide [1]. According to the annual report of the European Renal Association–European Dialysis and Transplant Association (ERA-EDTA), in 2016, in Europe and the Mediterranean Sea countries, renal replacement therapy (RRT) was initiated in 83,311 patients for ESRD, with an incidence of 121 per million inhabitants [1]. Most patients (84%) started RRT with hemodialysis (HD), and then approximately two-thirds of patients with ESRD continued to receive HD [1]. During HD, patients are exposed to continuous and repetitive changes in fluid volume that may lead to acute and chronic hemodynamic stress [2]. It is known that intravascular fluid depletion due to the start of the dialysis session may lead to hypovolemia and, as a consequence, to hypotension and impairment of organ perfusion, including the central nervous system [3]. On the other hand, sodium and fluid accumulation occur during the interdialytic phase, which may lead to hypertension and cerebrovascular complications [3]. Consequently, HD patients are more likely than the general population to have white matter hyperintensities on magnetic resonance imaging (MRI), a marker for small-vessel ischemic disease, and are therefore at higher risk for future stroke or cognitive decline [4,5]. It is known that HD patients are at increased risk of developing cognitive problems such as mild cognitive impairment (MCI), i.e., mild, noticeable deterioration of cognitive functions, and dementia [6,7,8]. Up to 80% of HD patients aged 55 and older have been shown to have moderate to severe chronic cognitive impairment (CI) [6,7]. Moreover, MCI and vascular dementia were most commonly observed in patients undergoing HD [9,10,11]. However, data on depression, anxiety, and various domains of cognitive impairment and their association with clinical parameters in dialyzed patients are very limited or even non-existent [12]. The aim of this study was to investigate the relationship between HD and MCI or suspected dementia and the influence of other factors on this cognitive decline. Moreover, all patients were screened for symptoms of depression and anxiety.

## 2. Materials and Methods

In this cross-sectional study, 74 of 95 consecutive patients (78%) undergoing HD, who agreed to participate in the study, were recruited at a single HD center between 1 August 2022 and 31 December 2022. Patients were included if they had ESRD, were on ambulatory HD, and were 18 years of age or older, and this occurred after consultation with the treatment team. Moreover, we included clinically stable patients without infectious disease in the last 8 weeks, decompensated heart, liver failure, psychiatric or neurodegenerative disorders, and delirium. Twenty-one patients were excluded. Three patients declined to participate in the study. Additionally, we excluded 9 patients due to the language barrier and another 9 patients due to physical disabilities such as hearing and vision impairment, limb paresis, and prior severe cognitive problems. Cognitive impairment (MCI and suspected dementia) and anxiety and depression were evaluated using the Addenbrooke Cognitive Test III (ACE III) and the Hospital Anxiety and Depression Scale (HADS), respectively. Demographic data (age, gender, years of education) and medical history (primary renal disease, duration of dialysis, comorbidities, results of laboratory tests) were obtained from hospital clinic records. The local Ethical Committee approved the study protocol (approval number KB/81/2022). Written informed consent was obtained from all participants.

### 2.1. Cognitive Function Evaluation

The A version of the ACE III test was used to evaluate cognitive function, including attention, memory, verbal fluency, language, and visuospatial abilities with a score ranging from 0 to 100 points. HD patients were assessed during the first hours of the HD session and at about the same time by one of two researchers. According to Kaczmarek et al., cut-off points of ≤88 and 82 points for MCI, ≤81 points for a high probability of dementia (suspected dementia), and ≥89 points for intact cognition were established [13]. ACE III is a reliable and validated screening test with high sensitivity and specificity in detecting cognitive problems [13]. At a threshold of 81, the sensitivity for detecting of suspected dementia was 84% and the specificity was 86% [13].

### 2.2. Anxiety and Depression Evaluation

The HAD Scale was used to measure anxiety and depression. It is a self-report questionnaire designed to screen clinically significant anxiety and depression, and it takes 2 to 5 min to complete [14]. The questionnaire consists of 7 questions separately for anxiety and depression, in which respondents are asked how they felt in the past week, with a total score between 0 and 21, where higher scores indicate more severe depression and anxiety. Cut-off points ≥ 8 points were set separately for anxiety and depression [14]. The HAD scale has overall good psychometric properties and performs well in screening for symptom severity and prevalence of anxiety and depression [15].

### 2.3. Statistical Analysis

Statistical analysis was performed with use of IBM SPSS Statistics 26. The statistical methods used in the following analysis took into account the small size of the groups compared (groups with suspected dementia, MCI, and with normal cognition). Only the size of the normal cognition group was on the edge of the applicability of statistical methods using normality of distribution (31 cases), while the other groups consisted of significantly fewer cases. Hence, no assumptions were made about the normality of the distributions from which the random samples considered were drawn.

The Kruskal–Wallis test [16] was used to compare the distributions of the continuous parameters in the compared populations. For the assessment of diagnostic ability of a parameter as a binary classifier, the receiver operating characteristics (ROC) curve was used [17]. To compare nominal/categorical variables, Cramér’s V measure was used, which indicates the strength of association between such types of variables [18]. 

## 3. Results

Seventy-four adult patients were included in the study. The demographic and clinical characteristics of the subjects are summarized in Table 1. 

**Figure 1:**
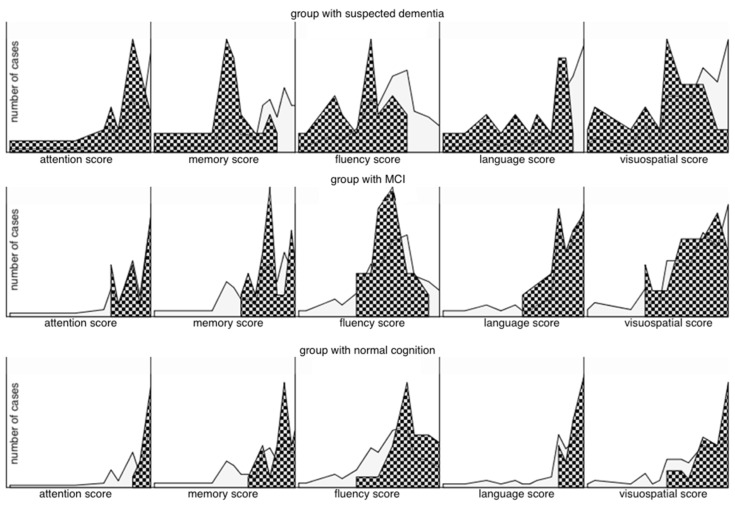
Histograms of the scores of the test in individual cognitive domains (attention, memory, fluency, language, visuospatial abilities) in groups with suspected dementia (**top row**), MCI (**middle row**), and normal cognition (**bottom row**)—meshed areas. Histograms of test results for all subjects (for all groups)—light grey area.

The mean age was 65.69 ± 14 years, and 27 individuals (36%) were women. Most of the patients had secondary education with a mean of 13.64 years of education (Table 1). In the majority of patients, the etiology of kidney disease was unknown, and diabetes mellitus was the second most common cause. The mean HD duration was 36.62 months. The mean ACE III score was 83 ± 13.4 (Table 1). Patients with MCI and suspected dementia accounted for 27% and 31% of all subjects, respectively (Table 1). The prevalence of MCI was higher in opposite to the prevalence of suspected dementia, which was lower in HD patients under the age of 65 (38% vs. 11.5%). In HD patients over the age of 65, the trend was reversed (21% vs. 42%). Histograms of the test results shown by individual cognitive domains and by patient group (Figure 1) indicate that in the group of patients with suspected dementia, all cognitive functions (according to ACE-III test) had significantly lower values compared to cognitively unimpaired and MCI patients. 

**Figure 2:**
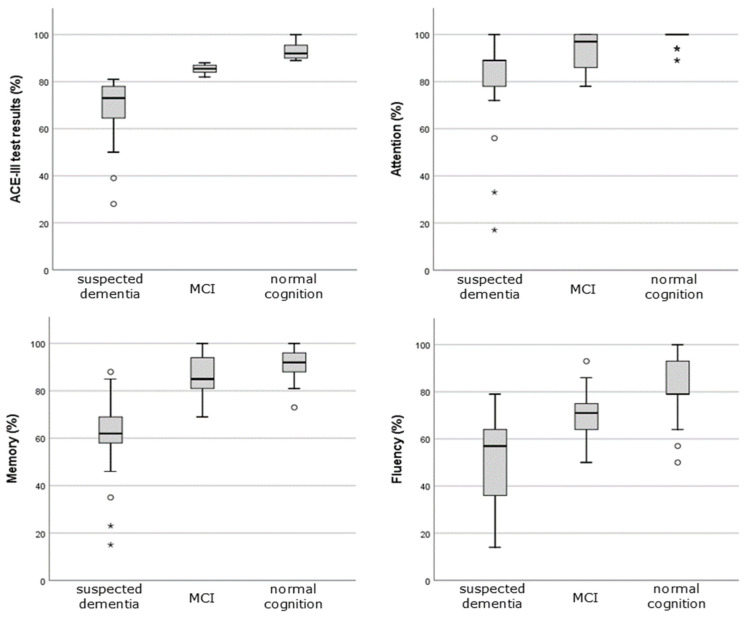

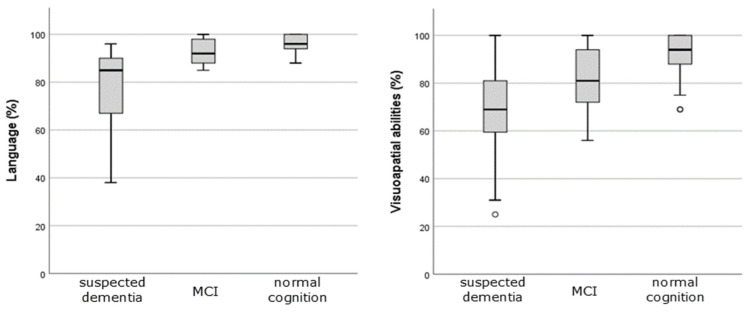
Kruskal–Wallis test of independence for ACE III test results (attention, memory, fluency, language, and visuospatial abilities). In all cases, the significancy was 0.000 across categories of interpretation (suspected dementia, MCI, normal cognition). Additional symbols used in boxplots: °, *—potential outliers and extreme values respectively.

**Figure 3:**
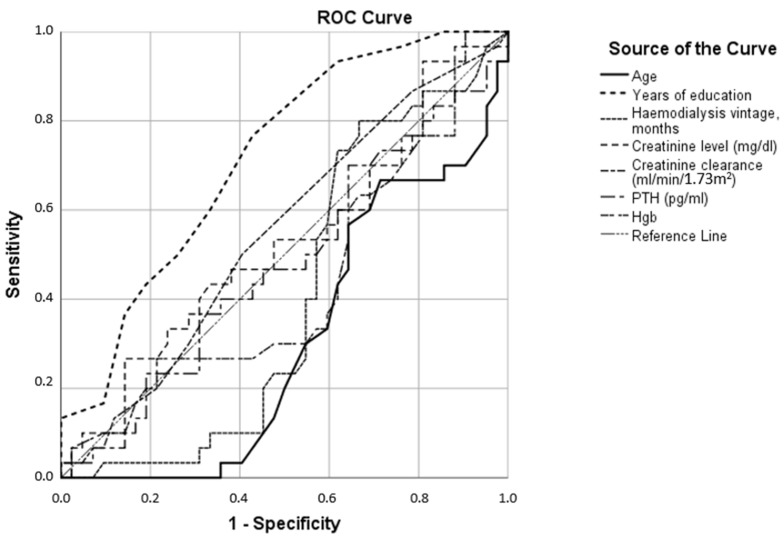
Receiver operating characteristics of various parameters (age, years of education, hemodialysis vintage, creatinine level, creatinine clearance, parathyroid hormone (PTH), and hemoglobin levels (Hgb)) as a cognitive impairment binary classifier.

For example, the area marked with a grid occupied the entire range of possible test results for the attention domain in the group with suspected dementia (upper left panel of Figure 1), while it was increasingly cumulative around the maximum values of the test when we considered patients with MCI or even more with normal cognition (bottom left panel of Figure 1). A similar situation was noticeable in all other cognitive domains, with the fluency as most impaired. Differences in test results between MCI and patients without cognitive decline were also statistically significant (significance of difference between median values of each group in all cases was 0.000) (Figure 2), with the smallest differences obtained in the domains of memory, attention, and language (Figure 2). The effect of parameter values on the possibility of using them for binary classification into CI and the normal cognition group was examined. The ROC curves plotted for the parameters age, years of education, hemodialysis vintage, creatinine level, creatinine clearance, parathyroid hormone (PTH), and hemoglobin levels (Hgb) are shown in Figure 3. The largest sensitivity and specificity for classifying CI and normal cognition are shown by years of education (area under ROC curve AUC = 0.71) and inversely by age (AUC = 0.31). The other parameters—creatinine levels, creatinine clearance, hemodialysis duration, PTH, and hemoglobin levels (the corresponding ROC curves were much closer to the reference line)—provided AUC values closer to 0.5, i.e., indicating a more random classification quality. 

**Figure 4:**
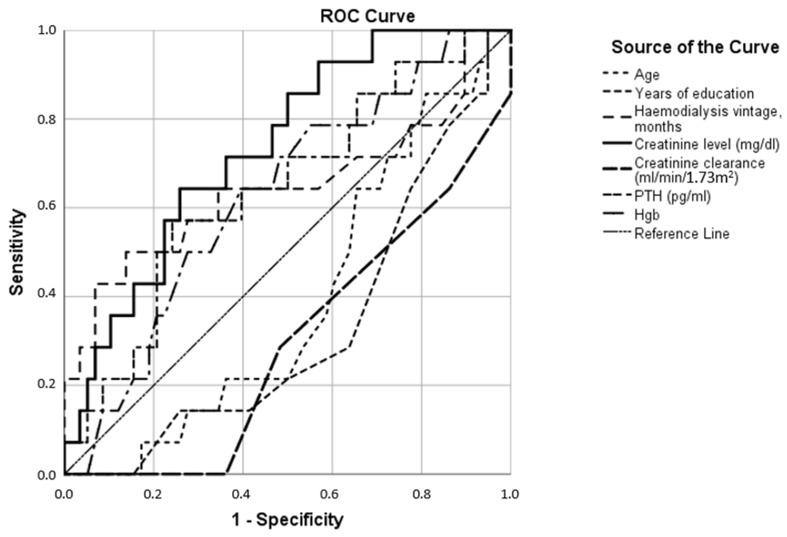
Receiver operating characteristics of various parameters (age, years of education, hemodialysis vintage, creatinine level, creatinine clearance, parathyroid hormone (PTH), and hemoglobin levels (Hgb)) as anxiety binary classifiers.

**Figure 5:**
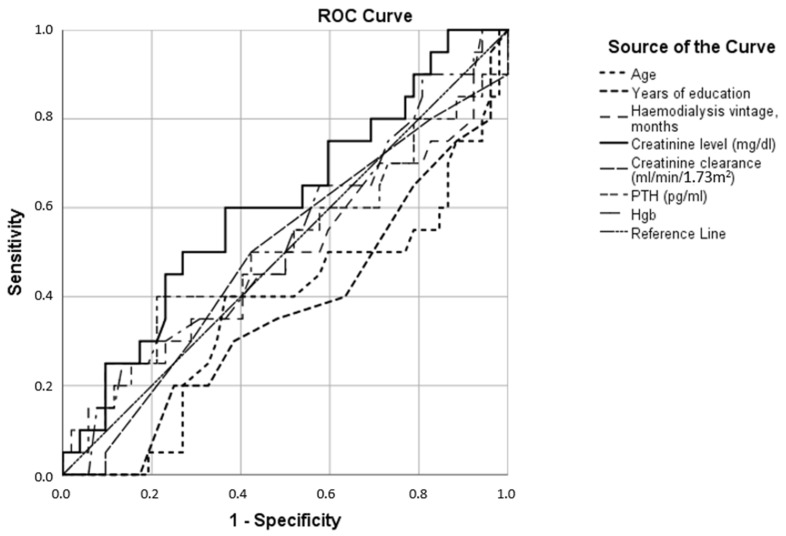
Receiver operating characteristics of various parameters (age, years of education, hemodialysis vintage, creatinine level, creatinine clearance, parathyroid hormone (PTH), and hemoglobin levels (Hgb)) as a depression binary classifiers.

When the same parameters were treated as classifying anxiety and depression, it turned out that anxiety was more related to these parameters (Table 2, Figure 4) than depression (Figure 5), wherein all parameters provided ROC curves close to the reference line (random classification).

In addition, younger and less educated patients were more likely to experience anxiety (Figure 4); at the same time, no factors contributing to the occurrence of depressive symptoms were found, with values of AUC close to 0.5 (Figure 5).

We also found that 28% and 22% of patients had depression and anxiety, respectively, on the screening tests, and mixed symptoms of depression and anxiety were 15%. Patients with anxiety symptoms had higher levels of endogenous creatinine, PTH, and hemoglobin, as well as decreased creatinine clearance (Figure 4, Table 2).

Finally, there was no significant effect of nominal (categorical) variables: gender, cardiovascular disease, hypertension, diabetes mellitus, smoking, depression, and anxiety on the occurrence of MCI and suspected dementia (Table 3), where Cramér’s V statistics are shown. 

## 4. Discussion

In the present study, we found a high rate of CI in patients undergoing HD therapy, with 31% of patients classified as MCI and 27% of patients with suspected dementia. Our results are consistent with previous studies showing a high prevalence of CI in HD patients ranging from 25% to 80% [6,7,9,19,20]. In a large multicenter study, 71.1% of 676 HD patients had cognitive problems in at least one cognitive domain [6]. In another two studies, the prevalence levels of CI among 108 an 408 HD patients were 25% [19] and 75%% [7], respectively. Conversely, in a study of 181 HD patients, 26.1% were classified as MCI and 31.5% as suspected dementia [20]. Patterns of cognitive decline in our study showed that all cognitive domains, including verbal fluency, memory, language, attention, and visuospatial, were impaired in HD patients with suspected dementia and MCI. Of these, the verbal fluency domain was the most severely affected. Verbal fluency tasks in screening tests reflect the domains of executive function and are a useful and validated tool for its assessment [21]. Our findings that executive function was poor in those undergoing HD are consistent with previous studies [9,22]. Of the 338 HD patients, 41% were found to be impaired in executive function (scored ≥ 2.0 SD below the population norm) [9]. Another study evaluating 80 HD patients found that 38% of them had impaired executive function [23]. In another study of 314 HD patients, executive function impairment was found in 40% (scored 1.5 SD below the population norm) [22]. Explicit executive function deficits characterize vascular dementia (VaD), while memory deficits may not be apparent in the early phase VaD [24]. Progressive memory impairment, mainly short-term memory loss, is the hallmark of Alzheimer’s disease [25]. Patients undergoing HD are at higher risk of vascular disease, which may be the primary cause of CI in this group of people [22]. In addition, an MRI of the brain showed that HD patients had more severe white matter changes and brain atrophy as a result of cerebrovascular events compared to controls without kidney disease, which may further lead to cognitive decline [26]. Although CI may be associated with traditional vascular risk factors such as cerebrovascular disease, hypertension, diabetes mellitus, or smoking and renal-specific risk factors such as anemia, hyperparathyroidism, chronic inflammation, and oxidative stress [11,27,28], we did not confirm such correlations. One explanation may be that HD patients are at increased risk for subclinical cerebrovascular diseases such as asymptomatic brain infarction, cerebral microbleeds, and white matter lesions, which may gradually lead to MCI and dementia [27]. Additionally, in our study, dementia was more common in people over the age of 65, which is in line with the global trend indicating that the average level of cognitive functioning decreases with age [29]. We also showed that more highly educated patients performed better on cognitive tests. Finally, we found that the prevalence levels of depressive and anxiety symptoms were 28% and 22%, respectively, and that of mixed depressive and anxiety symptoms was 15%. The results of our study are similar to the previous studies [12,30]. We showed that younger patients were more prone to anxiety symptoms. One explanation may be concerns about social and occupational constraints, treatment-related complications, vascular access surgeries, and the expected length of treatment. In addition, we found that more highly educated patients were less likely to have anxiety symptoms, and this may be explained by the association of education levels with a better functional understanding of chronic disease or medical terminology used by healthcare professionals. Moreover, patients on HD for longer periods experienced greater anxiety. We also found that high endogenous creatinine, PTH, and hemoglobin levels and decreased creatinine clearance were positively correlated with anxiety symptoms, which was consistent with previous studies [31,32]. Guerra et al. showed that high levels of creatinine were positively associated with high stress levels and psychological distress [31]. In another study, anxiety was reported in a higher portion of patients with primary hyperparathyroidism than in controls [32]. In all our patients, hyperparathyroidism was secondary to metabolic bone disease. Although there is a growing awareness about depression and anxiety in patients undergoing HD, which are common complications of chronic diseases, there is little research on this topic. In our study, we focused on the comprehensive assessment of cognitive function together with depression and anxiety. In addition, we aimed to assess the relation between studied parameters and clinical and biochemical characteristics of the HD patients. 

There are some limitations associated with this study. Patients were tested at the day of the dialysis session. The dialysis unit consisted of several treatment areas, and this can be a noisy environment that could lead to distractions during the cognitive assessment. In addition, as an invasive therapy, HD may cause anxiety in patients, which may impact test results. Moreover, there was an overrepresentation of male subjects and a relatively small sample size. Therefore, larger studies are needed to better describe the characteristics and association with levels of cognitive impairment.

## 5. Conclusions

Identification of HD patients with CI, depression, and anxiety symptoms is important for improving quality of life, adherence to dietary and fluid restrictions, dialysis, and pharmacotherapy regimens, as well as for providing professional health care, including mental health specialists and neurological care. Early diagnosis of cognitive and psychological problems also allows for the implementation of appropriate therapy, including non-pharmacological interventions, and in some patients, it may shorten the time to be waitlisted for transplantation. Therefore, it seems reasonable to implement screening programs in dialysis centers. 

## Figures and Tables

**Table 1:** Demographic characteristics of the study group.

	All (n = 74)	Normal Cognition (n = 31)	MCI (n = 20)	Suspected Dementia (n = 23)
Age, years, mean ± SD (range)	65.69 ± 13.9 (28, 94)	60.19 ± 13.242 (28, 75)	64.3 ± 12.96 (31, 86)	74.3 ± 11.57 (50, 94)
Female, n (%)	27 (36)	10 (37)	6 (22)	11 (41)
Years of education, mean ± SD (range)	13.64 ± 3.4 (7, 23)	15.23 ± 3.19 (10, 23)	13 ± 3.18 (8, 18)	12.04 ± 2.98 (7, 18)
Hemodialysis vintage, months, mean ± SD (range)	36.62 ± 47.5 (1, 297)	22.55 ± 23 (1, 120)	56 ± 68.52 (1, 297)	38.74 ± 45.76 (1, 180)
Cardiovascular disease, n (%)	31 (41.9)	11 (35.5)	8 (40)	12 (52.2)
Hypertension, n (%)	63 (85.1)	28 (90.3)	15 (75)	20 (87)
Diabetes mellitus, n (%)	24 (32.4)	10 (32.3)	3 (15)	11 (47.8)
Nonsmoking, n (%)	57 (77)	24 (77.4)	15 (75)	18 (78.3)
ACE-III test results, % ± SD, (range)	83.04 ± 13.4 (28, 100)	92.81 ± 3.26 (89, 100)	85.35 ± 1.84 (82, 88)	67.87 ± 14.04 (28, 81)
Attention, % ± SD (range)	90.69 ± 14.5 (17, 100)	98.32 ± 3.34 (89, 100)	92.25 ± 9.01 (78, 100)	79.04 ± 19.66 (17, 100)
Fluency, % ± SD (range)	68.93 ± 19.2 (14, 100)	81.94 ± 12.29 (50, 100)	69.15 ± 11.5 (50, 93)	51.22 ± 18.29 (14, 79)
Language, % ± SD (range)	89.2 ± 13.3 (38, 100)	96.26 ± 4.49 (88, 100)	92.15 ± 6.97 (73, 100)	77.13 ± 17 (38, 96)
Memory, % ± SD (range)	80.23 ± 18.0 (15, 100)	91.55 ± 6.99 (73, 100)	85.45 ± 6.64 (69, 100)	60.43 ± 18.27 (15, 88)
Visuospatial abilities, % ± SD (range)	81.95 ± 17.7 (25, 100)	92.48 ± 9.7 (69, 100)	80.7 ± 14.01 (56, 100)	68.83 ± 19.96 (25, 100)
Anxiety, n (%)	16 (21.6)	5 (16.1)	5 (25)	6 (26)
Depression, n (%)	21 (28.4)	7 (22.6)	6 (30)	8 (34.8)
Creatinine clearance (ml/min/1.73 m^2^), mean ± SD (range)	6.32 ±2.8 (3, 18)	6.45 ± 2.85 (3, 18)	5.6 ± 2.84 (4, 17)	6.78 ± 2.61 (3, 13)
Creatinine level (mg/dL), mean ± SD (range)	8.11 ± 2.3 (3.29, 14.79)	8.3 ± 2.45 (3.29, 14.79)	8.88 ± 2.07 (3.56, 12.74)	7.19 ± 2.2 (4.13, 11.01)
Hemoglobin (g/dL), mean ± SD (range)	10.757 ±1.4 (7.4, 13.8)	10.62 ± 1.29 (8.3, 13)	10.7 ± 0.88 (8.6, 11.9)	11 ± 1.78 (7.4, 13.8)
Parathyroid hormone (pg/mL), mean ± SD (range)	450.65 ± 382.8 (42.8, 2484.0)	412.53 ± 299.98 (42.8, 1208)	482.22 ± 331.82 (86.3, 1126)	473.95 ± 517.71 (70.8, 2484)

**Table 2:** Values of area under ROC curve AUC for classification of anxiety using various parameters (age, years of education, hemodialysis vintage, creatinine level, creatinine clearance, parathyroid hormone (PTH), and hemoglobin levels (Hgb)).

Anxiety Test Result Variables	AUC
Age	0.390
Years of education	0.328
Hemodialysis vintage, months	0.646
Creatinine level (mg/dL)	0.735
Creatinine clearance (mL/min/1.73 m^2^)	0.297
Parathyroid hormone (pg/mL)	0.644
Hemoglobin (g/dL)	0.627

**Table 3:** Impact of various factors on cognitive impairment as a result of Cramér’s V [0;1] statistics.

	Cramer’s V	
	Value	Significance (=Chi-Squared)
Gender	0.159	0.391
Education category	0.273	0.087
Depression	0.116	0.606
Anxiety	0.114	0.620
Hypertension	0.178	0.310
Diabetes mellitus	0.267	0.072
Vascular disease	0.145	0.460
Smoking	0.096	0.852

## Data Availability

All data generated or analyzed during this study are included in this article. Further enquiries can be directed to the corresponding author.
